# Divergent Roles of HIF-1α and HIF-2α in Embryonic Development and Early Pregnancy

**DOI:** 10.3390/ijms27031593

**Published:** 2026-02-06

**Authors:** Hossam H. Shawki, Asmaa Y. Ammar, Mohamed Mansour, Fatma M. Minisy

**Affiliations:** 1 Department of Comparative and Experimental Medicine, Nagoya City University Graduate School of Medical Sciences, Nagoya 467-8601, Japan; 2 National Gene Bank of Egypt, ARC, Giza 12619, Egypt; 3 Biotechnology Department, Animal Health Research Institute, ARC, Giza 12619, Egypt; asmaa_ammar86@yahoo.com; 4 Department of Pathology, National Research Centre, Cairo 12622, Egypt

**Keywords:** hypoxia, HIF-1α, HIF-2α, endometrial, menstrual, implantation, decidualization, angiogenesis

## Abstract

Physiological hypoxia is a defining feature of early pregnancy, coordinating menstrual repair, implantation, decidualization, placental development, and fetoplacental adaptation. Hypoxia-inducible factors, HIF-1α and HIF-2α, act as master regulators of these processes by sensing oxygen tension and orchestrating cellular responses in metabolism, angiogenesis, immune regulation, and tissue remodeling. Although structurally related, HIF-1α and HIF-2α exhibit distinct spatial and temporal functions across reproductive stages. Embryonic HIF-1α is primarily involved in early embryonic development, whereas embryonic HIF-2α is required for later developmental stages. Furthermore, maternal HIF-1α acts early in pregnancy, coordinating metabolic adaptation, endometrial regeneration, decidualization, angiogenic expansion, placental organization, and maternal immune tolerance. In contrast, maternal HIF-2α regulates epithelial breakdown, trophoblast invasion, implantation mechanics, and vesicle-mediated trafficking. Mouse genetics demonstrate that disruption of either isoform leads to non-redundant defects in reproductive success, from failed implantation to placental insufficiency and fetal lethality. Pathological hypoxia or aberrant HIF signaling drives pregnancy disorders including preeclampsia, fetal growth restriction, recurrent pregnancy loss, and heavy menstrual bleeding. Defining the distinct roles of HIF-1α and HIF-2α supports the development of therapies targeting hypoxia-responsive pathways in infertility and obstetric disease.

## 1. Introduction

Oxygen availability is a key regulator of cellular fate during embryonic development and pregnancy. Hypoxia, defined as reduced oxygen at the tissue or cellular level, is not merely a stress condition but an essential physiological signal in early gestation. During menstruation and early pregnancy, oxygen tension in the endometrium falls to approximately 1–3% O_2_ in the luminal epithelium and subepithelial stroma, creating a controlled hypoxic environment that supports endometrial repair, embryo implantation, decidualization, trophoblast plasticity, immune tolerance, and placental development [1,2]. The reproductive system is particularly sensitive to oxygen fluctuations due to its high metabolic activity, hormonal cycles, and dependence on precise vascular regulation. While spatially and temporally restricted physiological hypoxia supports pregnancy progression, excessive duration, altered spatial distribution, or mistimed HIF activation disrupts reproductive function and contributes to disease. Pathological hypoxia, arising from oxidative stress, inadequate uteroplacental perfusion, or defective vascular remodeling, contributes to pregnancy disorders such as preeclampsia, fetal growth restriction, and early pregnancy loss [3,4,5].

The primary mediators of the cellular response to hypoxia are the hypoxia-inducible factors (HIFs). HIFs are transcription factors that sense low oxygen tension and activate the expression of hypoxia-responsive genes [6]. By regulating these targets, HIFs orchestrate essential physiological processes such as metabolism, angiogenesis, and cell survival [7,8]. Structurally, HIFs consist of an oxygen-sensitive α subunit (HIF-1α, HIF-2α, or HIF-3α) and a constitutively expressed β subunit HIF-1β (ARNT). Under normoxic conditions, the α subunit is hydroxylated by prolyl hydroxylase domain (PHD) proteins, recognized by the Von Hippel–Lindau (VHL) complex, and degraded by the proteasome. In contrast, hypoxia suppresses PHD activity, allowing HIF-α stabilization, nuclear translocation, and dimerization with HIF-1β. This complex then binds to hypoxia-responsive elements (HREs) and activates the transcription of target genes [9].

Although HIF-1α and HIF-2α share structural similarity and regulate overlapping hypoxia-responsive targets, their roles in pregnancy are temporally and spatially distinct. HIF-1α primarily drives metabolic adaptation, tissue repair, and immune programming, whereas HIF-2α orchestrates vesicular trafficking and vascular-matrix architecture. These non-redundant mechanisms produce sharply contrasting phenotypes when either isoform is disrupted. Understanding these dynamics not only clarifies fundamental reproductive biology but also opens avenues for therapeutic strategies and biomarker development in conditions such as preeclampsia, recurrent pregnancy loss, and infertility.

This review aims to summarize current knowledge on HIF-1α and HIF-2α as orchestrators of reproductive hypoxia, with a focus on their temporal expression, molecular targets, and distinct functions. By distinguishing their roles in embryonic development, menstruation, implantation, decidualization, placentation, and fetoplacental adaptation, we provide a comprehensive framework for understanding HIF signaling in early pregnancy. While previous reviews have summarized hypoxic signaling in placental cells and its association with miscarriage, the present review emphasizes the divergent, non-redundant roles of HIF-1α and HIF-2α across multiple reproductive stages and integrates genetic and mechanistic evidence into a stage-resolved framework.

## 2. Structural Divergence Between HIF-1α and HIF-2α

Hypoxia-inducible factor-1α (HIF-1α) and hypoxia-inducible factor-2α (HIF-2α) are paralogous α-subunits that heterodimerize with HIF-1β (ARNT) to form the core hypoxia-inducible transcriptional complex. Both proteins share a modular architecture that includes a basic helix–loop–helix (bHLH) domain, Per-Arnt-Sim domains (PAS-A and PAS-B), an oxygen-dependent degradation domain (ODDD), and N- and C-terminal transactivation domains (N-TAD and C-TAD) [10,11]. HIF-1α expression is ubiquitous, whereas its paralog HIF-2α shows a more restricted tissue distribution [12,13,14,15,16]. Despite this conserved framework, key sequence divergences endow each isoform with distinct regulatory and physiological roles.

**Figure 1:**
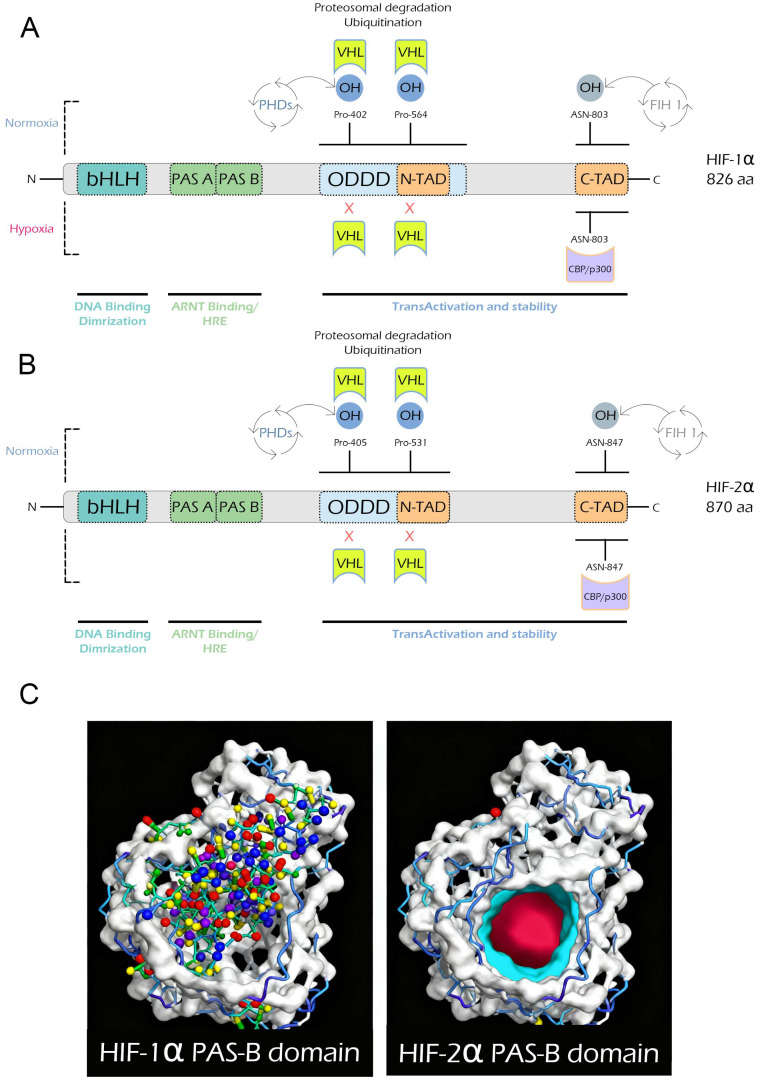
**Domain architecture and regulatory differences between HIF-1α and HIF-2α.** (**A**) Human HIF-1α (826 aa). The protein comprises a basic helix–loop–helix (bHLH) DNA-binding domain, Per-Arnt-Sim domains (PAS-A and PAS-B) mediating dimerization with ARNT, an oxygen-dependent degradation domain (ODDD), and N- and C-terminal transactivation domains (N-TAD and C-TAD). Under normoxia, proline residues (Pro402, Pro564) are hydroxylated by prolyl hydroxylases (PHDs), allowing recognition by the von Hippel–Lindau (VHL) complex and proteasomal degradation. Concurrently, hydroxylation of Asn803 by factor-inhibiting HIF 1 (FIH 1) prevents binding of the transcriptional co-activators CREB-binding protein (CBP) and E1A-binding protein p300 (p300). Under hypoxia, hydroxylation is inhibited, allowing CBP/p300 recruitment, HIF-1α stabilization, and transcriptional activation of hypoxia-responsive genes. (**B**) Human HIF-2α (870 aa). HIF-2α shares the same modular design but differs in its hydroxylation sites (Pro405, Pro531) and C-TAD asparagine residue (Asn847). Its PAS-B domain harbors a well-defined ligand-binding pocket absent in HIF-1α, providing a structural basis for isoform-specific regulation and selective inhibition. (**C**) A representative image of the PAS-B domains of HIF-1α and HIF-2α, revealing the structural basis for ligand selectivity. The HIF-2α PAS-B domain (right) contains a well-defined internal cavity that forms a druggable pocket, lined by small residues, creating sufficient space for small-molecule ligand binding. In contrast, the HIF-1α PAS-B domain (left) lacks such a pocket; bulkier residues occupy the same region, collapsing the internal space and blocking ligand access.

Human HIF-1α is composed of 826 amino acids, while HIF-2α contains 870 (Figure 1). Each protein contains an N-terminal bHLH domain that mediates DNA binding, PAS-A and PAS-B domains that mediate dimerization with HIF-1β/ARNT and cofactor binding, an ODDD that regulates protein stability via prolyl hydroxylation and VHL-mediated degradation (Pro402 and Pro564 in HIF-1α; Pro405 and Pro531 in HIF-2α), and N- and C-terminal transactivation domains that interact with transcriptional co-activators such as CBP/p300 [17,18,19,20]. Hydroxylation of a conserved asparagine residue within the C-TAD (Asn803 in HIF-1α and Asn847 in HIF-2α) blocks co-activator recruitment and fine-tunes transcriptional output. Although these elements are shared, sequence identity between the isoforms varies considerably; the bHLH and PAS domains are highly conserved (>50% identity), whereas the N- and C-TADs diverge substantially, explaining their differential transcriptional specificity [14,21].

Post-translational control of both isoforms depends on oxygen-dependent hydroxylation. Proline hydroxylation within the ODDD permits pVHL recognition and proteasomal degradation under normoxia, while hydroxylation of the C-TAD asparagine by factor-inhibiting HIF (FIH) prevents recruitment of transcriptional co-activators [21]. Although this regulatory logic is shared, sequence divergence surrounding these motifs influences cofactor preferences and transcriptional selectivity.

A particularly striking structural distinction resides in the PAS-B domain. HIF-2α contains a well-defined ligand-binding cavity that is absent or poorly formed in HIF-1α. This feature has been therapeutically exploited, enabling the development of small-molecule inhibitors that selectively block HIF-2α activity [22]. The most notable example is the FDA–approved HIF-2α inhibitor Belzutifan, used to treat clear-cell renal cell carcinoma [23,24]. Beyond pharmacological relevance, this structural divergence contributes to differences in dimer stability and protein half-life.

Under normoxic conditions, both HIF-1α and HIF-2α are rapidly hydroxylated at conserved proline residues within their ODDD, ubiquitinated by pVHL, and degraded via the proteasome, thereby preventing dimerization with HIF-1β/ARNT and transcriptional activation [25,26]. Under hypoxia, proline and asparagine hydroxylation are inhibited, leading to stabilization, nuclear accumulation, dimerization with HIF-1β/ARNT, and binding to hypoxia-responsive elements (HREs). At this stage, their structural and sequence differences become functionally decisive, dictating unique gene targets, tissue specificity, and co-activator interactions.

Although they share a conserved modular design, the distinct features of HIF-1α and HIF-2α particularly the differences in ODDD prolyl hydroxylation sites, differences in C-TAD sequence context, presence of the druggable PAS-B pocket in HIF-2α, and distinct transcriptional networks; underlie their non-redundant physiological roles and contrasting therapeutic relevance.

## 3. Non-Redundant Developmental Roles of HIF-1α and HIF-2α

Insights from mouse genetics provide the clearest demonstration that HIF-1α and HIF-2α orchestrate non-redundant developmental programs, although the extent to which these mechanisms are conserved in human embryogenesis remains an active area of investigation. Despite their structural homology and shared dimerization partner (HIF-1β/ARNT), these transcription factors act sequentially during embryogenesis, reflecting distinct roles in hypoxic adaptation and organogenesis.

Global deletion of HIF-1α in mice leads to embryonic lethality around embryonic day (E)10.5–11.0 due to cardiac, vascular, and neural defects [27,28,29]. Beginning at E8.5, mutant embryos exhibit severe growth retardation, neural tube closure failure, cephalic cystic degeneration, branchial arch hypoplasia, myocardial hyperplasia with pericardial effusion, and vascular disorganization. Detailed analyses reveal extensive mesenchymal and neural crest cell death in the cranial region preceding vascular regression, leading to loss of pericyte support and collapse of endothelial networks [27,29]. The yolk-sac vasculature fails to form a branched capillary network, and cardiac chambers become obliterated, causing circulatory failure.

At the molecular level, HIF-1α deficiency disrupts glycolytic and hypoxia-responsive gene activation such as phosphoglycerate kinase (PGK), despite paradoxically elevated *Vegfa* mRNA driven by glucose deprivation rather than HIF-1α activity [29]. Thus, embryonic lethality results not from VEGF deficiency but from failure of cellular adaptation to hypoxia, leading to metabolic collapse, mesenchymal apoptosis, vascular regression, and multi-organ malformation by mid-gestation.

In contrast, deletion of HIF-2α causes lethality at later stages (E12.5–E16.5), depending on genetic background [30,31,32,33]. While early vasculogenesis proceeds normally, vascular remodeling is severely compromised, resulting in endothelial sheet formation, hemorrhage, and vascular leakage in the yolk sac and embryonic tissues [31]. Loss of HIF-2α also impairs catecholamine synthesis within the organ of Zuckerkandl, producing cardiac dysfunction characterized by bradycardia, reduced norepinephrine levels, and circulatory failure [30]. Administration of DOPS, a norepinephrine precursor, partially rescues embryonic lethality, confirming the essential role of HIF-2α in catecholamine homeostasis.

A subset of *Hif-2α^−^/^−^* mice survive to term but die shortly after birth. HIF-2α regulates VEGF expression in alveolar type II pneumocytes, which is crucial for converting glycogen stores into surfactant and for alveolar expansion [32]. These mutants die from respiratory distress syndrome (RDS) due to impaired lung maturation. Neutralization of VEGF signaling reproduces this phenotype, whereas intra-amniotic VEGF administration improves survival. By crossing mice on different genetic backgrounds, Scortegagna et al. (2003, 2005) were able to obtain a small fraction of viable *Hif-2α*-mutant adult mice [33,34]. These mice exhibit severe anemia, pancytopenia, hypocellular bone marrow, reduced erythropoietin expression, growth retardation, hepatomegaly, cardiac hypertrophy, and intolerance to hypoxia.

Double deletion of *Hif-1α* and *Hif-2α* results in lethality before E8.5, substantially earlier than either single knockout, and is characterized by a complete absence of placental vasculogenesis, underscoring their cooperative yet non-overlapping developmental functions [35]. Functional substitution experiments further confirm this divergence. Replacement of HIF-1α with HIF-2α cDNA at the HIF-1α locus causes death before E7.5, indicating that the two proteins are not functionally interchangeable despite shared DNA-binding motifs [36].

Together, both isoforms are indispensable for normal embryonic progression, but each executes its role at distinct developmental windows and in distinct cellular contexts, affirming their evolutionarily specialized programs of hypoxic adaptation.

## 4. Menstrual Breakdown and Repair Are Programed by HIF-1α

During the menstrual cycle, which is tightly regulated by ovarian hormones and classically divided into proliferative, secretory, and menstrual phases, the endometrium undergoes cyclical breakdown followed by rapid, scarless regeneration. The successful transition from the menstrual to the proliferative phase depends on effective repair of damaged spiral arterioles and restoration of epithelial integrity, a process critically governed by hypoxia.

**Figure 2:**
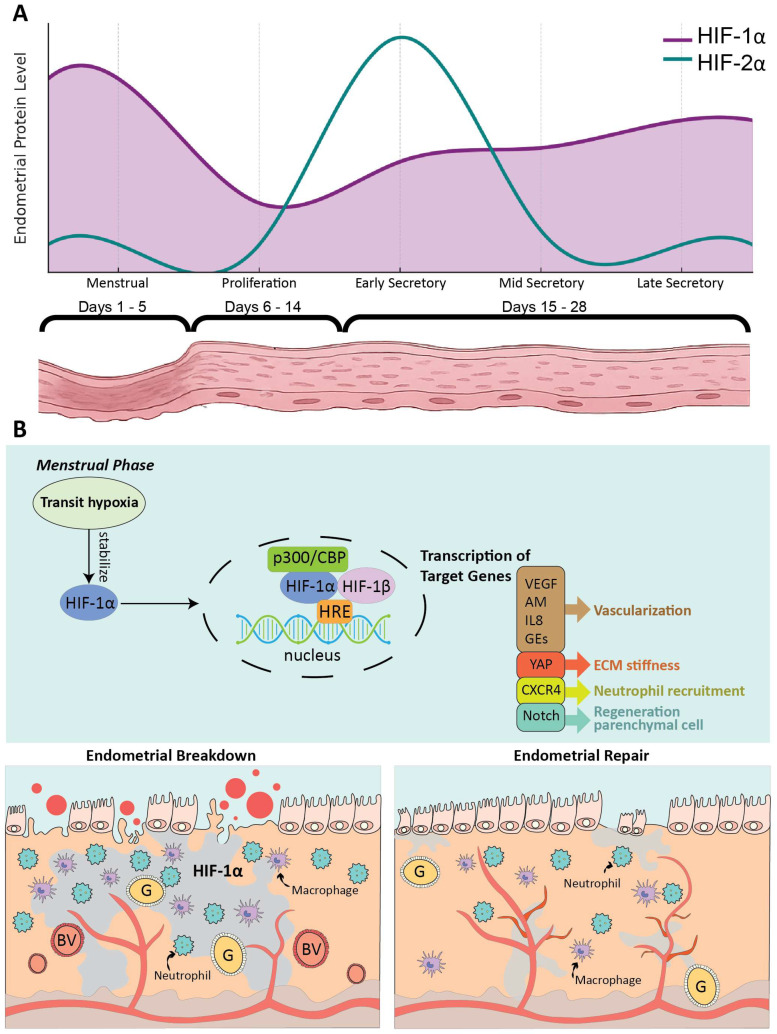
**Hypoxia orchestrate menstrual breakdown and repair through HIF-1α-dependent gene activation.** (**A**) Schematic representation of relative HIF-1α and HIF-2α protein levels across the menstrual cycle, conceptually redrawn from Western blot densitometry reported by Maybin et al. (Nat Commun, CC-BY 4.0) [1]. HIF-1α expression peaks during the menstrual phase, coinciding with endometrial breakdown and the onset of tissue repair, indicating its essential role in hypoxia-driven regeneration. In contrast, HIF-2α expression is largely absent during the actual repair stage. The lower panel illustrates the histological changes in the endometrium across menstrual, proliferative, and secretory phases. (**B**) During the peri-menstrual phase, local hypoxia stabilizes HIF-1α, enabling its dimerization with HIF-1β and recruitment of the co-activator p300/CBP to form a transcriptionally active complex that binds hypoxia-responsive elements (HREs) in the nucleus. This activation induces the expression of several downstream targets critical for tissue repair. These include VEGF, AM, IL-8, and GEs, which promote vascularization; YAP, which regulates extracellular-matrix stiffness and mechanotransduction essential for structural integrity; CXCR4, which directs neutrophil infiltration to clear debris and initiate regeneration; and Notch signaling, which drives proliferation and differentiation of endometrial progenitor cells to restore the epithelial lining. VEGF, vascular endothelial growth factor; AM, adrenomedullin; IL-8, interleukin-8; GEs, glycolytic enzymes; YAP, yes-associated protein; CXCR4, C-X-C chemokine receptor 4; G, glands; BV, blood vessel.

In women with normal menstrual bleeding, peri-menstrual hypoxia stabilizes HIF-1α, which drives angiogenesis and repair by inducing VEGF, IL-8, adrenomedullin (AM), CXCR4, and glycolytic enzymes (GEs), thereby promoting vascular remodeling, re-epithelialization, and immune cell recruitment, in addition to the self-renewal of endometrial mesenchymal stem-like cells through Notch signaling (Figure 2) [1,37,38,39]. Experimental models confirm that blocking hypoxia by exposure to 75% O_2_, partial genetic loss of HIF-1α, or pharmacologic inhibition with echinomycin impairs VEGF induction, endothelial branching, and tissue repair [1]. Conversely, stabilizing HIF-1α with prolyl hydroxylase enzyme inhibitors such as DMOG or roxadustat restores vascular function and accelerates regeneration. In vitro, silencing HIF-1α in endometrial epithelial cells reduces VEGF secretion and disrupts endothelial network formation, a defect rescued by exogenous VEGF [1].

Beyond angiogenesis, HIF-1α contributes to immune homeostasis. Insufficient HIF-1α activity or impaired immune-cell recruitment delays epithelial shedding and repair, whereas CXCR4–mediated neutrophil infiltration facilitates tissue regeneration [40]. At the structural level, HIF-1α integrates with redox-sensitive pathways to regulate yes-associated protein (YAP), maintaining extracellular-matrix stiffness and mechanotransduction essential for endometrial integrity [41]. Clinically, reduced HIF-1α protein but not mRNA in women with heavy menstrual bleeding (HMB) suggests defective stabilization and post-translational regulation rather than transcriptional loss, whereas women with obesity exhibit paradoxically elevated HIF-1α and target-gene expression, also associated with impaired repair [42,43].

By contrast, HIF-2α appears to play a minor, indirect role in endometrial repair. Its expression is limited to the secretory phase preceding menstruation and is largely absent during the repair stage [1]. Human and mouse studies indicate that inappropriate elevation of HIF-2α before menstruation may slightly delay repair and contribute to heavier bleeding, whereas reducing HIF-2α may modestly accelerate repair and reduce blood loss, though these effects do not reach statistical significance [44].

Collectively, menstrual hypoxia is a physiological cue that stabilizes HIF-1α to drive angiogenesis, epithelial regeneration, immune cell recruitment, and stem/progenitor renewal essential for timely endometrial repair. HIF-2α appears to contribute to vascular remodeling and preparation for implantation rather than direct endometrial repair, although its precise mechanisms in the non-pregnant endometrium particularly on tissue breakdown and regeneration remain incompletely understood and warrant further investigation. Pharmacological activation of HIF-1α therefore represents a promising non-hormonal, fertility-preserving therapeutic strategy for heavy menstrual bleeding, although translation to clinical practice will require careful validation in human studies.

## 5. HIF-2α Is a Master Regulator During Implantation and Receptivity

During embryo implantation, the maternal uterus orchestrates a complex interplay of hypoxia-driven signaling and steroid hormone responsiveness that ensures reproductive success [45]. HIF-1α is primarily expressed in uterine epithelial cells during the peri-implantation period and extends into the stromal compartment as implantation progresses [16]. In contrast, HIF-2α displays a more dynamic and tightly regulated expression pattern. It is selectively induced in subluminal stromal cells at the time of peri-implantation, with expression increasing during implantation in response to the combined influences of estrogen signaling, progesterone priming, and reduced oxygen availability [16].

HIF-1α broadly supports vascular permeability and angiogenesis, and is positively associated with endometrial receptivity [46,47,48]. More specifically, HIF-1α upregulates VEGF and drives glycolytic reprogramming in stromal cells [47,48]. These processes enhance vascular density, optimize energy metabolism, and improve oxygen delivery, creating a uterine environment conducive to implantation. Clinical studies align with these findings, showing that reduced HIF-1α expression correlates with poor microvessel density and diminished uterine receptivity [48,49].

Conditional deletion of uterine HIF-1α in mice causes subfertility with smaller litter sizes. Although Matsumoto et al. [50] have not clarify whether the defect originates during implantation, earlier work suggests a role in promoting uterine receptivity. However, most studies of HIF-1α have focused on its roles in angiogenesis, vascular permeability, and stromal transformation rather than direct involvement in embryo invasion or epithelial remodeling [46,47,48,49]. Therefore, the mechanistic basis of its function remains incompletely defined. Its loss produces partial but underexplored defects, implying that HIF-1α acts mainly as a facilitator, possibly improving conditions for implantation without being absolutely indispensable.

Unlike HIF-1α, stromal HIF-2α is indispensable for the physical mechanics of embryo invasion. Conditional deletion of HIF-2α in the uterus results in complete infertility. Embryos attach to the luminal epithelium but fail to penetrate, leaving the epithelial barrier intact and stromal remodeling programs inactive [50]. This phenotype underscores the unique role of HIF-2α in coordinating epithelial detachment, extracellular matrix remodeling, and angiogenesis.

**Figure 3:**
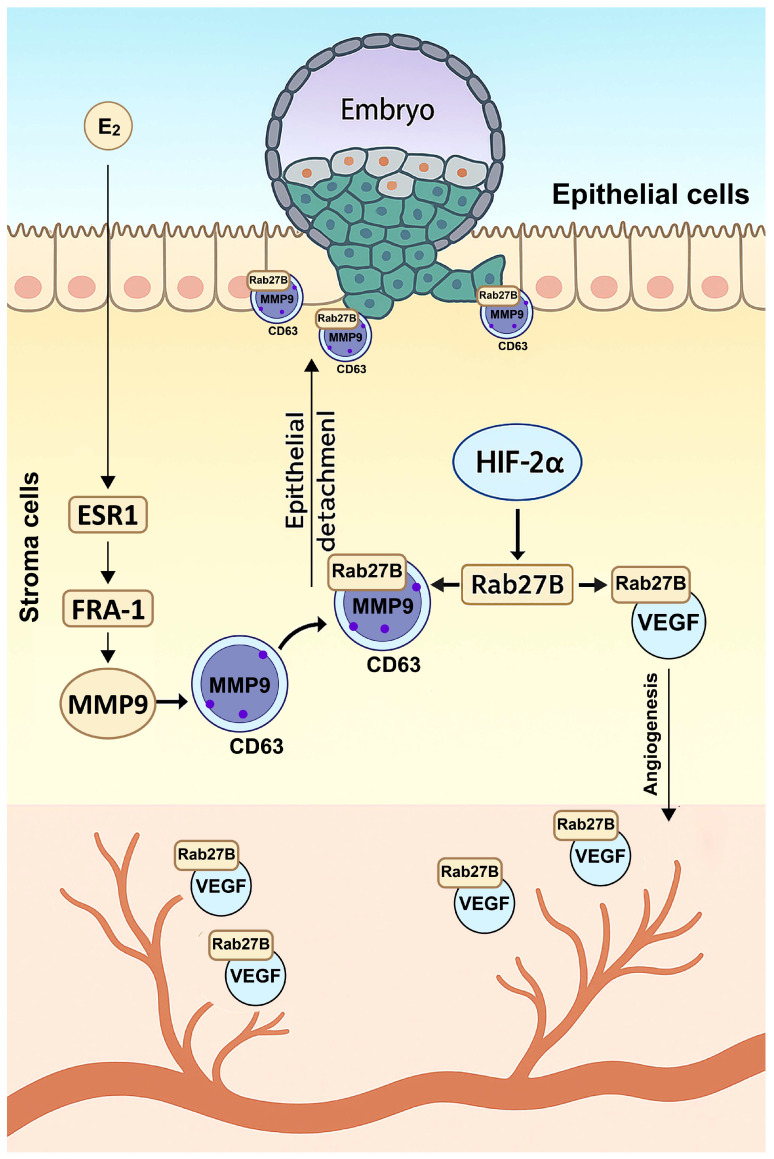
**HIF-2α controls stromal vesicle trafficking during embryo implantation and decidualization.** Estrogen (E_2_) enters stromal cells and activates ESR1, which induces the transcription factor FRA-1 to upregulate *Mmp-9* expression. HIF-2α, in parallel, activates Rab27B to regulate vesicular trafficking. Rab27B directs the movement of CD63-marked secretory vesicles carrying MMP-9 toward the stromal cell surface, where they undergo exocytosis into the subepithelial region. The localized release of MMP-9 facilitates epithelial detachment, enabling blastocyst penetration. In addition, the HIF-2α–Rab27B pathway promotes vesicular release of VEGF granules from stromal to endothelial cells, stimulating angiogenic network formation and vascular perfusion essential for early pregnancy during implantation and decidualization. ESR1, estrogen receptor alpha; FRA-1, Fos-related antigen 1; MMP-9, matrix metallopeptidase 9; Rab27B, Ras-related protein Rab-27B; CD63, tetraspanin CD63; VEGF, vascular endothelial growth factor.

Mechanistically, implantation depends on the combined actions of estrogen-driven transcription and HIF-2α-regulated secretory trafficking (Figure 3). Estrogen signaling through estrogen receptor alph (ESR1) induces the transcription factor FRA-1, which upregulates *Mmp-9* expression in differentiating stromal cells [51]. HIF-2α does not influence *Mmp-9* transcription but instead induces *Rab27B* expression in stroma cells during implantation, which directs the movement of secretory granules carrying MMP-9 [52]. These granules are marked by CD63, a tetraspanin that serves as a scaffold for MMP-9–containing vesicles. Under HIF-2α control, Rab27B mobilizes CD63–positive vesicles to the stromal cell surface, where they undergo exocytosis into the subepithelial region. This coordinated mechanism ensures both sufficient synthesis of MMP-9 under estrogen–FRA-1 control and its spatially targeted release under HIF-2α regulation. The localized secretion of MMP-9 drives basement membrane degradation and epithelial remodeling at the implantation site, enabling blastocyst penetration [52].

Beyond MMP-9 trafficking, the HIF-2α-Rab27B pathway governs vesicular release of VEGF granules from stromal to endothelial cells, promoting angiogenic network formation and vascular perfusion that sustain early pregnancy [52]. In parallel, HIF-2α maintains LIF–STAT3 signaling, which is vital for embryo attachment. Deletion of HIF-2α markedly reduces LIF expression and abolishes STAT3 activation at the implantation site, preventing luminal epithelial breakdown [50,52]. Consistent with these defects, the expression of genes mediating extracellular matrix remodeling and vascular adaptation including *Mmps*, *Vegfa*, *Lox*, and *Adm* is sharply downregulated in HIF-2α deficient stroma [50]. Hormonal or cytokine supplementation with progesterone or LIF fails to rescue implantation failure, underscoring the non-redundant and irreplaceable role of HIF-2α in maternal remodeling [50].

Taken together, uterine HIF-1α contributes primarily supportive metabolic and angiogenic functions that facilitate receptivity, and its deletion causes subfertility, although the precise stage of failure remains incompletely defined. In contrast, genetic deletion studies show that loss of HIF-2α produces a direct implantation block, establishing it as the non-redundant driver of epithelial remodeling and embryo invasion. These findings position stromal HIF-2α as the master regulator of implantation mechanics. It integrates estrogen-driven transcriptional programs with hypoxia-induced vesicular trafficking to coordinate epithelial barrier breakdown, stromal remodeling, and angiogenesis that together enable successful embryo invasion. However, the extent to which these mechanisms operate identically in human implantation remains incompletely resolved, highlighting an important area for future investigation.

## 6. HIF-1α and HIF-2α Are Required with Divergent Roles in Decidualization

As the embryo penetrates the uterine epithelium and reaches the stroma, extensive remodeling of the maternal tissue occurs, forming a specialized structure called the decidua. Decidualization is the differentiation of endometrial stromal cells into secretory cells that support embryo development and placental formation. Impaired decidualization has been recognized as a major factor underlying pregnancy disorders such as preeclampsia, miscarriage, and preterm labor [53]. Both HIF-1α and HIF-2α expression are upregulated in the uterus during this process; although HIF-2α shows markedly stronger expression [16,54].

**Figure 4:**
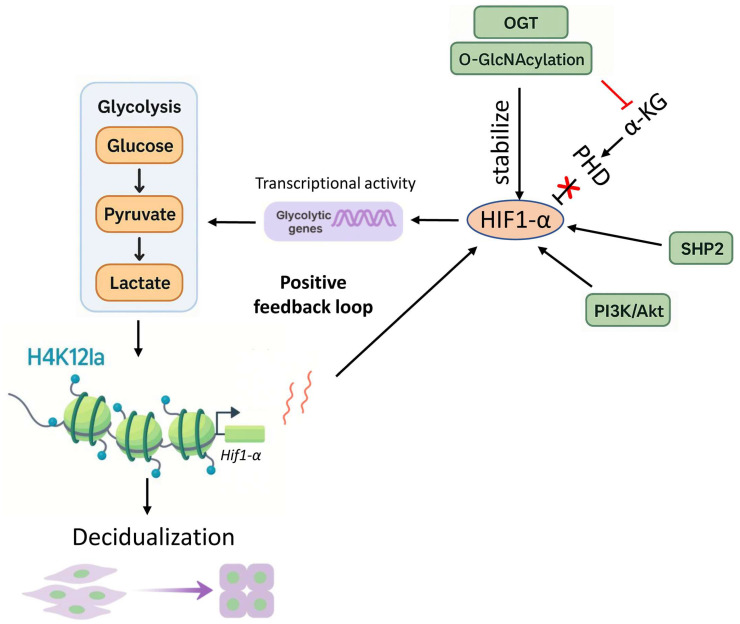
**HIF-1α mediates glycolytic regulation to drive decidualization.** Glycolysis converts glucose to pyruvate and lactate, which enhances *Hif-1α* transcriptional activity through histone H4 lysine 12 lactylation (H4K12la). Multiple pathways stabilize HIF-1α, including OGT-mediated O-GlcNAcylation that inhibits α-KG-dependent prolyl hydroxylation (PHD), as well as SHP2 and PI3K/Akt signaling. Stabilized HIF-1α establishes a positive feedback loop that further amplifies glycolysis and lactate-driven chromatin remodeling to promote stromal cell differentiation and decidualization. H4K12la, histone H4 lysine 12 lactylation; α-KG, α-ketoglutarate; OGT, O-GlcNAc transferase; PI3K, phosphoinositide 3-kinase; Akt, protein kinase B.

HIF-1α regulates glycolytic activity in endometrial stromal cells, and its stability and expression are essential for decidualization (Figure 4) [54,55]. Bo Li et al. [54] demonstrated that O-GlcNAc transferase (OGT), which mediates protein O-GlcNAcylation, stabilizes HIF-1α and thereby enhances glycolysis during decidualization. Pharmacological inhibition of either OGT or HIF-1α markedly decreased decidualization in human endometrial stromal cells (HESCs), as evidenced by reduced expression of decidualization markers prolactin (PRL) and IGFBP1, and by reduced decidual tissue mass in vivo following HIF-1α inhibitor treatment in pregnant mice. Importantly, restoring HIF-1α stability with a prolyl hydroxylase (PHD) inhibitor partially rescued PRL expression suppressed by OGT inhibition, confirming that O-GlcNAcylation promotes decidualization in part through HIF-1α stabilization.

In another study, enhanced glycolytic activity and increased local lactate production within the endometrium induced histone lactylation, specifically at histone H4 lysine 12 lactylation (H4K12la) [55]. This modification promotes HIF-1α expression and establishes an H4K12la–HIF-1α–glycolysis positive feedback loop. Experimental inhibition of histone lactylation impairs decidualization, both under physiological conditions and in in vivo and in vitro models [55].

Recent findings also demonstrate that protein tyrosine phosphatase 2 (SHP2) is highly expressed in decidualized cells and governs the progression of decidualization. SHP2 acts as a pivotal upstream regulator of HIF-1α signaling during human endometrial stromal cell differentiation [56]. By directly interacting with and stabilizing HIF-1α, SHP2 enhances its transcriptional activity, leading to the upregulation of key glycolytic genes such as *ENO3*, *PKM2*, *ALDOC*, and *GLUT1*. Knockdown or inhibition of SHP2 significantly reduced HIF-1α and its downstream targets, as well as lactate production in decidual cells. This metabolic shift highlights how the SHP2–HIF-1α axis integrates metabolic and signaling cues to drive glycolysis, histone lactylation, and gene expression reprogramming essential for stromal cell differentiation.

Furthermore, Zuo et al. demonstrated that progesterone activates the PI3K/Akt pathway, leading to increased HIF-1α expression in decidual stromal cells [57]. HIF-1α subsequently upregulates PKM2 and MCT4, enhancing lactate production and secretion [57]. The released lactate is taken up by surrounding proliferative stromal cells via MCT1, promoting their growth. Conversely, HIF-1α knockdown reduces both lactate production and cell proliferation, underscoring that HIF-1α coordinates lactate generation and utilization in the decidual microenvironment during early pregnancy [57]. Together, these studies identify HIF-1α as a key regulator coordinating glycolytic metabolism and lactate-mediated signaling to drive proper decidualization.

Although early work suggested that HIF-2α directly drives the decidualization program, an integrated analysis of mouse and human datasets reveals a more nuanced mechanism in which HIF-2α supports decidualization indirectly by controlling the secretory and vascular architecture of the uterus [50,52,58]. In the initial report, uterine deletion of HIF-2α disrupted decidual–ovarian crosstalk and caused infertility accompanied by defective decidualization. Loss of HIF-2α suppressed prolactin-related factors (Prl3c1 and Prlr), key decidual luteotrophins required to maintain corpus luteum function and progesterone production [50]. However, subsequent studies using artificial decidualization models demonstrated that core transcriptional regulators of decidualization (Pgr, Hand2, Wnt4, CEBPβ) remain largely intact in HIF-2α-deficient mouse uterus. Instead, the dominant phenotype is the loss of the vascular network surrounding the implantation chamber [52].

Mechanistically, HIF-2α acts as a regulator of Rab27B-dependent secretory trafficking (Figure 3), directing the docking and exocytosis of vesicles enriched in VEGFA as well as extracellular vesicles (EVs) containing metabolic transporters such as GLUT1 [52,58]. These vesicles are taken up by neighboring stromal and endothelial cells, where they stimulate proliferation, metabolic support, and angiogenic remodeling. Human studies further support this model, decidualizing HESCs secrete EVs via a conserved HIF-2α–Rab27B pathway, and these EVs carry signaling proteins and growth modulators that enhance stromal differentiation and promote endothelial expansion [58]. These findings indicate that HIF-2α synchronizes glucose metabolism, secretory vesicle trafficking, and endothelial proliferation, a process indispensable for building a functional decidua. Thus, the decidualization defect initially described by Matsumoto et al. [50] likely reflects secondary consequences of impaired stromal secretion and angiogenesis, which disrupt progesterone-dependent uterine–ovarian communication, rather than a primary block in stromal differentiation.

Evidence consistently supports HIF-1α-dependent metabolic remodeling as a core driver of stromal differentiation, whereas mouse genetics and human EV studies increasingly suggest HIF-2α is required to construct the decidual microenvironment via Rab27B-dependent secretion/angiogenesis rather than to directly specify the canonical decidual transcriptional program.

## 7. HIF-1α Is Pivotal for Placentation and Feto-Maternal Immune Tolerance

HIF-1α and HIF-2α are expressed in the human placenta, but their abundance is tightly linked to the hypoxic milieu of early gestation. During the first trimester, both isoforms localize to syncytiotrophoblasts, villous cytotrophoblasts, and fetal endothelial cells, aligning with the physiological hypoxia that supports early placental development [59,60,61,62,63,64]. HIF-1α shows dynamic regulation, reaching its highest expression between 7 and 10 weeks when oxygen levels are lowest, followed by a progressive decline as maternal blood flow to the intervillous space is established [64]. In contrast, HIF-2α expression remains relatively constant throughout gestation [61,64].

Genetic mouse models have provided direct evidence that maternal HIF-1α is required for normal placental morphogenesis. Conditional deletion of *Hif-1α* in maternal tissues at E8.5 results in defects in both the maternal decidua and fetal-derived placental layers [65]. Placenta of the *Hif-1α* cKO shows reduced decidual size, decreased numbers and Tpbpa-positive trophoblast cells in the decidua, and marked alterations in the junctional zone, including expansion and premature migration of glycogen trophoblast cells towards the decidua [65,66]. The labyrinth of cKO exhibits reduced numbers and size of Gcm1-positive syncytiotrophoblast progenitors and an underdeveloped branching architecture, consistent with impaired syncytial differentiation and morphogenesis [65,67,68]. Functionally, these placental abnormalities render fetuses highly vulnerable to oxygen deprivation after mid-gestation, with growth restriction, abnormal cardiac morphology, and increased fetal lethality by E15.5 [65]. Thus, adequate maternal HIF-1α signaling is essential for establishing a properly patterned decidua and junctional zone, and for building a labyrinth capable of sustaining fetoplacental gas exchange.

HIF-1α also orchestrates a maternal immune landscape through recruitment of decidual immune cells and establish the immunosuppression at the maternal-fetal interface [2,69]. HIF-1α activity in decidual natural killer (dNK) cells promotes transcription of *Igf1* and *Gdf15*, factors that enhance trophoblast migration and anchoring [70]. Loss of maternal HIF-1α leads to depletion of dNK cells and impaired trophoblast invasion, producing defected placentation [65]. These findings underscore that HIF-1α-dependent immune–trophoblast crosstalk is a critical upstream regulator of early placental formation. Furthermore, macrophages are similarly triggered by HIF-1α-dependent signals to promote the formation of materno-fetal immune tolerance [71,72]. HIF-1α activation in both decidual stromal cells and decidual macrophages, enhancing secretion of CCL2 and promoting CCR2-mediated macrophage accumulation and their immune status [73]. In addition, HIF-1α plays a key role in regulating the expansion and survival of myeloid-derived suppressor cells (MDSCs), which contribute to maternal–fetal tolerance. Myeloid-specific deletion of *Hif1*α prevents the accumulation of MDSCs in the pregnant uterus, increases their apoptosis, and results in early pregnancy loss [74].

Maternal HIF-2α supports placentation through vascular–matrix remodeling rather than direct immune programming. It permits trophoblast invasion and chorio-decidual formation by maintaining angiogenic balance and tissue mechanics, positioning it as an indirect regulator of immune homeostasis [50,52]. Unlike HIF-1α, which directly shapes dNK cells, macrophages, and MDSCs, HIF-2α mainly creates the permissive vascular and extracellular environment that enables tolerogenic immune function. Although hypoxia-driven HIF-2α can favor M2-like macrophage behavior [75,76], genetic models show that loss of maternal HIF-2α leads primarily to defects in vascular remodeling and epithelial detachment rather than overt immune tolerance failure.

Taken together, available genetic evidence supports a central role for maternal HIF-1α in coordinating placental morphogenesis and immune tolerance, but the relative weighting of immune-mediated versus trophoblast-intrinsic mechanisms remains incompletely resolved. Because most mechanistic insight derives from mouse models with distinct immune and placental architecture, determining which HIF-driven immune programs are conserved in human pregnancy remains an important open question.

## 8. Summary and Future Directions

Hypoxia is not merely a byproduct of fluctuating uteroplacental perfusion; it is a tightly controlled physiological signal that shapes every stage of early pregnancy. HIF-1α and HIF-2α act as the primary molecular regulators of this signal, but their functions are distinctly partitioned. HIF-1α supports endometrial regeneration, angiogenesis and decidualization through metabolic reprogramming, while orchestrating placentation and maternal immune tolerance. In contrast, HIF-2α governs the physical mechanics of implantation by regulating vesicular trafficking. The temporal partitioning of HIF-1α and HIF-2α functions across early pregnancy is summarized in Table 1. Together, these non-redundant transcription factors ensure successful menstruation, implantation, decidualization, and placentation. When dysregulated, whether through pathological hypoxia or impaired HIF signaling, these processes fail, resulting in heavy menstrual bleeding, infertility, recurrent miscarriage, preeclampsia, and fetal growth restriction.

Distinguishing physiological from pathological hypoxia is essential in interpreting HIF signaling during early pregnancy. Physiological hypoxia is spatially restricted and transient, typically reaching ~1–3% O_2_ in defined endometrial and placental niches, where it drives tightly timed HIF activation required for implantation, decidualization, and placental development. In contrast, pathological hypoxia associated with pregnancy disorders appears to reflect dysregulation of otherwise physiological pathways. Current evidence suggests that aberrant HIF activation in conditions such as preeclampsia is best explained by excessive duration, altered spatial distribution, and/or inappropriate developmental timing of hypoxic signaling rather than activation of fundamentally distinct pathways.

Despite major progress in defining tissue-specific and temporal functions of HIF isoforms, several key questions remain unresolved. A deeper investigation into how HIF-1α and HIF-2α coordinate metabolic–immune interactions at the maternal–fetal interface is needed, particularly in human tissues where immune architecture diverges from mouse models. The precise contribution of HIF-2α to menstrual breakdown and repair also requires clarification. Uterine HIF-1α deletion causes subfertility, but the mechanistic basis and timing of implantation defects remain unclear. Moreover, clinical translation demands improved biomarkers that distinguish physiological from pathological hypoxia in real time.

While mouse models have provided invaluable insights into the distinct roles of HIF-1α and HIF-2α, it is important to acknowledge their limitations when extrapolating findings to human reproductive biology. Fundamental differences exist in placental structure, immune-cell composition, and the temporal dynamics of pregnancy between mice and humans. For example, although the murine placenta is hemochorial, it contains distinct trophoblast lineages and vascular architecture compared to the human placenta. Likewise, the regulation of decidual immune populations, particularly NK cells and macrophages, is not identical across species, meaning that some immune-modulatory mechanisms observed in mice may not translate directly to human pregnancy. Thus, while mouse genetics clearly demonstrate non-redundant HIF functions, distinguishing conserved hypoxic signaling pathways from species-specific adaptations remains essential for therapeutic development and biomarker identification.

Future research should integrate genetic, single-cell, spatial-omics, and EV-based approaches to map cell-specific HIF networks throughout early pregnancy. Targeted pharmacologic strategies, such as selective HIF-2α inhibitors and hypoxia-mimetic HIF-1α stabilizers, hold promise but must be carefully tailored to reproductive timing due to the distinct stage-dependent functions of each isoform. Prioritizing cell-type-specific HIF mapping, experimental testing of EV-mediated signaling in human tissues, and development of biomarkers that distinguish physiological from pathological hypoxia represents an achievable near-term roadmap. Supported by spatial single-cell technologies, organoid models, and EV profiling, these directions will accelerate translation of hypoxia biology into precision therapies that restore reproductive oxygen homeostasis without compromising maternal or fetal health.

## Figures and Tables

**Table 1:** Temporal partitioning of HIF-1α and HIF-2α functions across stages of early pregnancy.

Developmental Window	Dominant HIF Isoform	Primary Biological Function
Early embryogenesis (up to E10.5–E11.0)	HIF-1α	Metabolic adaptation and early vascular organization
Late embryogenesis (E12.5–E16.5 to postnatal)	HIF-2α	Vascular remodeling and organ maturation
Peri-menstrual phase	HIF-1α	Endometrial breakdown and repair
Peri-implantation phase	HIF-2α	Epithelial detachment and invasion mechanics
Early decidual phase	HIF-1α (metabolic)/ HIF-2α (secretory)	Stromal differentiation and vascular remodeling
Placentation	HIF-1α dominant	Placentation and immune tolerance

## Data Availability

No new data were created or analyzed in this study. Data sharing is not applicable to this article.

## References

[1] Maybin J.A., Murray A.A., Saunders P.T.K., Hirani N., Carmeliet P., Critchley H.O.D. (2018). Hypoxia and Hypoxia Inducible Factor-1α Are Required for Normal Endometrial Repair during Menstruation. Nat. Commun..

[2] Zhao H., Wong R.J., Stevenson D.K. (2021). The Impact of Hypoxia in Early Pregnancy on Placental Cells. Int. J. Mol. Sci..

[3] Tal R. (2012). The Role of Hypoxia and Hypoxia-Inducible Factor-1alpha in Preeclampsia Pathogenesis. Biol. Reprod..

[4] Ishii T., Miyazawa M., Takanashi Y., Tanigawa M., Yasuda K., Onouchi H., Kawabe N., Mitsushita J., Hartman P.S., Ishii N. (2014). Genetically Induced Oxidative Stress in Mice Causes Thrombocytosis, Splenomegaly and Placental Angiodysplasia That Leads to Recurrent Abortion. Redox Biol..

[5] Tong W., Giussani D.A. (2019). Preeclampsia Link to Gestational Hypoxia. J. Dev. Orig. Health Dis..

[6] Dengler V.L., Galbraith M.D., Espinosa J.M. (2014). Transcriptional Regulation by Hypoxia Inducible Factors. Crit. Rev. Biochem. Mol. Biol..

[7] Rey S., Luo W., Shimoda L.A., Semenza G.L. (2011). Metabolic Reprogramming by HIF-1 Promotes the Survival of Bone Marrow-Derived Angiogenic Cells in Ischemic Tissue. Blood.

[8] Ong S.-G., Lee W.H., Theodorou L., Kodo K., Lim S.Y., Shukla D.H., Briston T., Kiriakidis S., Ashcroft M., Davidson S.M. (2014). HIF-1 Reduces Ischaemia–Reperfusion Injury in the Heart by Targeting the Mitochondrial Permeability Transition Pore. Cardiovasc. Res..

[9] Hon W.-C., Wilson M.I., Harlos K., Claridge T.D.W., Schofield C.J., Pugh C.W., Maxwell P.H., Ratcliffe P.J., Stuart D.I., Jones E.Y. (2002). Structural Basis for the Recognition of Hydroxyproline in HIF-1α by pVHL. Nature.

[10] Wang G.L., Semenza G.L. (1995). Purification and Characterization of Hypoxia-Inducible Factor 1. J. Biol. Chem..

[11] Kinoshita K. (2004). Altered DNA Binding Specificity of Arnt by Selection of Partner bHLH-PAS Proteins. Nucleic Acids Res..

[12] Ema M., Taya S., Yokotani N., Sogawa K., Matsuda Y., Fujii-Kuriyama Y. (1997). A Novel bHLH-PAS Factor with Close Sequence Similarity to Hypoxia-Inducible Factor 1alpha Regulates the VEGF Expression and Is Potentially Involved in Lung and Vascular Development. Proc. Natl. Acad. Sci. USA.

[13] Flamme I., Fröhlich T., Von Reutern M., Kappel A., Damert A., Risau W. (1997). HRF, a Putative Basic Helix-Loop-Helix-PAS-Domain Transcription Factor Is Closely Related to Hypoxia-Inducible Factor-1α and Developmentally Expressed in Blood Vessels. Mech. Dev..

[14] Tian H., McKnight S.L., Russell D.W. (1997). Endothelial PAS Domain Protein 1 (EPAS1), a Transcription Factor Selectively Expressed in Endothelial Cells. Genes Dev..

[15] Semenza G.L. (2000). HIF-1: Mediator of Physiological and Pathophysiological Responses to Hypoxia. J. Appl. Physiol..

[16] Daikoku T., Matsumoto H., Gupta R.A., Das S.K., Gassmann M., DuBois R.N., Dey S.K. (2003). Expression of Hypoxia-Inducible Factors in the Peri-Implantation Mouse Uterus Is Regulated in a Cell-Specific and Ovarian Steroid Hormone-Dependent Manner. J. Biol. Chem..

[17] Jaakkola P., Mole D.R., Tian Y.-M., Wilson M.I., Gielbert J., Gaskell S.J., Kriegsheim A.V., Hebestreit H.F., Mukherji M., Schofield C.J. (2001). Targeting of HIF-α to the von Hippel-Lindau Ubiquitylation Complex by O_2_-Regulated Prolyl Hydroxylation. Science.

[18] Freedman S.J., Sun Z.-Y.J., Poy F., Kung A.L., Livingston D.M., Wagner G., Eck M.J. (2002). Structural Basis for Recruitment of CBP/P300 by Hypoxia-Inducible Factor-1α. Proc. Natl. Acad. Sci. USA.

[19] Kristan A., Debeljak N., Kunej T. (2021). Integration and Visualization of Regulatory Elements and Variations of the EPAS1 Gene in Human. Genes.

[20] Yan Q., Bartz S., Mao M., Li L., Kaelin W.G. (2007). The Hypoxia-Inducible Factor 2 *α* N-Terminal and C-Terminal Transactivation Domains Cooperate to Promote Renal Tumorigenesis In Vivo. Mol. Cell. Biol..

[21] Loboda A., Jozkowicz A., Dulak J. (2010). HIF-1 and HIF-2 Transcription Factors—Similar but Not Identical. Mol. Cells.

[22] Scheuermann T.H., Li Q., Ma H.-W., Key J., Zhang L., Chen R., Garcia J.A., Naidoo J., Longgood J., Frantz D.E. (2013). Allosteric Inhibition of Hypoxia Inducible Factor-2 with Small Molecules. Nat. Chem. Biol..

[23] Chan K.H., Li N., Lador R., Amsbaugh M., Gonzalez A., Cen P. (2024). Belzutifan, HIF-2α Inhibitor, and Clear Cell Renal Cell Carcinoma With Somatic Von-Hippel-Lindau Loss-of-Function Mutation. J. Investig. Med. High. Impact Case Rep..

[24] Wu X., Lazris D., Wong R., Tykodi S.S. (2025). Belzutifan for the Treatment of Renal Cell Carcinoma. Ther. Adv. Med. Oncol..

[25] Marxsen J.H., Stengel P., Doege K., Heikkinen P., Jokilehto T., Wagner T., Jelkmann W., Jaakkola P., Metzen E. (2004). Hypoxia-Inducible Factor-1 (HIF-1) Promotes Its Degradation by Induction of HIF-α-Prolyl-4-Hydroxylases. Biochem. J..

[26] Ferens F.G., Taber C.C., Stuart S., Hubert M., Tarade D., Lee J.E., Ohh M. (2024). Deficiency in PHD2-Mediated Hydroxylation of HIF2α Underlies Pacak-Zhuang Syndrome. Commun. Biol..

[27] Iyer N.V., Kotch L.E., Agani F., Leung S.W., Laughner E., Wenger R.H., Gassmann M., Gearhart J.D., Lawler A.M., Yu A.Y. (1998). Cellular and Developmental Control of O_2_ Homeostasis by Hypoxia-Inducible Factor 1α. Genes Dev..

[28] Ryan H.E. (1998). HIF-1alpha Is Required for Solid Tumor Formation and Embryonic Vascularization. EMBO J..

[29] Kotch L.E., Iyer N.V., Laughner E., Semenza G.L. (1999). Defective Vascularization of HIF-1α-Null Embryos Is Not Associated with VEGF Deficiency but with Mesenchymal Cell Death. Dev. Biol..

[30] Tian H., Hammer R.E., Matsumoto A.M., Russell D.W., McKnight S.L. (1998). The Hypoxia-Responsive Transcription Factor EPAS1 Is Essential for Catecholamine Homeostasis and Protection against Heart Failure during Embryonic Development. Genes Dev..

[31] Peng J., Zhang L., Drysdale L., Fong G.-H. (2000). The Transcription Factor EPAS-1/Hypoxia-Inducible Factor 2α Plays an Important Role in Vascular Remodeling. Proc. Natl. Acad. Sci. USA.

[32] Compernolle V., Brusselmans K., Acker T., Hoet P., Tjwa M., Beck H., Plaisance S., Dor Y., Keshet E., Lupu F. (2002). Loss of HIF-2α and Inhibition of VEGF Impair Fetal Lung Maturation, Whereas Treatment with VEGF Prevents Fatal Respiratory Distress in Premature Mice. Nat. Med..

[33] Scortegagna M., Morris M.A., Oktay Y., Bennett M., Garcia J.A. (2003). The HIF Family Member EPAS1/HIF-2α Is Required for Normal Hematopoiesis in Mice. Blood.

[34] Scortegagna M., Ding K., Zhang Q., Oktay Y., Bennett M.J., Bennett M., Shelton J.M., Richardson J.A., Moe O., Garcia J.A. (2005). HIF-2α Regulates Murine Hematopoietic Development in an Erythropoietin-Dependent Manner. Blood.

[35] Cowden Dahl K.D., Fryer B.H., Mack F.A., Compernolle V., Maltepe E., Adelman D.M., Carmeliet P., Simon M.C. (2005). Hypoxia-Inducible Factors 1α and 2α Regulate Trophoblast Differentiation. Mol. Cell. Biol..

[36] Covello K.L., Kehler J., Yu H., Gordan J.D., Arsham A.M., Hu C.-J., Labosky P.A., Simon M.C., Keith B. (2006). HIF-2α Regulates Oct-4: Effects of Hypoxia on Stem Cell Function, Embryonic Development, and Tumor Growth. Genes Dev..

[37] Zhang S., Chan R.W.S., Ng E.H.Y., Yeung W.S.B. (2022). Hypoxia Regulates the Self-Renewal of Endometrial Mesenchymal Stromal/Stem-like Cells via Notch Signaling. Int. J. Mol. Sci..

[38] Maybin J.A., Hirani N., Jabbour H.N., Critchley H.O.D. (2011). Novel Roles for Hypoxia and Prostaglandin E2 in the Regulation of IL-8 During Endometrial Repair. Am. J. Pathol..

[39] Maybin J.A., Battersby S., Hirani N., Nikitenko L.L., Critchley H.O.D., Jabbour H.N. (2011). The Expression and Regulation of Adrenomedullin in the Human Endometrium: A Candidate for Endometrial Repair. Endocrinology.

[40] Wang S., Chen X., Guo S., Zhou F., Zhang X., Lu C., Yang X., Wang Q., He B., Wang J. (2023). CXCR4, Regulated by HIF1A, Promotes Endometrial Breakdown via CD45+ Leukocyte Recruitment in a Mouse Model of Menstruation. Reprod. Biol..

[41] Zhang T., Wang Y., Wang Y., Liu C., Han C. (2022). Crosstalk between Extracellular Matrix Stiffness and ROS Drives Endometrial Repair via the HIF-1α/YAP Axis during Menstruation. Cells.

[42] Jain V., Chodankar R.R., Maybin J.A., Critchley H.O.D. (2022). Uterine Bleeding: How Understanding Endometrial Physiology Underpins Menstrual Health. Nat. Rev. Endocrinol..

[43] Reavey J.J., Walker C., Murray A.A., Brito-Mutunayagam S., Sweeney S., Nicol M., Cambursano A., Critchley H.O.D., Maybin J.A. (2021). Obesity Is Associated with Heavy Menstruation That May Be due to Delayed Endometrial Repair. J. Endocrinol..

[44] Martínez-Aguilar R., Rowley B.M., Walker C., Critchley H.O.D., Carmeliet P., Maybin J.A. (2025). Limiting Premenstrual Endometrial Hypoxia Inducible Factor 2 Alpha May Fine-Tune Endometrial Function at Menstruation. J. Clin. Endocrinol. Metab..

[45] Bagchi I.C., Bagchi M.K. (2024). Maternal–Fetal Mechanisms Underlying Adaptation to Hypoxia during Early Pregnancy. Trends Endocrinol. Metab..

[46] Semenza G.L. (2000). Expression of Hypoxia-Inducible Factor 1: Mechanisms and Consequences. Biochem. Pharmacol..

[47] Tsuzuki T., Okada H., Cho H., Tsuji S., Nishigaki A., Yasuda K., Kanzaki H. (2012). Hypoxic Stress Simultaneously Stimulates Vascular Endothelial Growth Factor via Hypoxia-Inducible Factor-1 and Inhibits Stromal Cell-Derived Factor-1 in Human Endometrial Stromal Cells. Hum. Reprod..

[48] Liang L., Yang Y., Yang L., Zhang X., Xu S., Liu Y., Wu X., Chao L. (2023). HIF-1α Is Positively Associated with Endometrial Receptivity by Regulating PKM2. J. Obs. Gynaecol..

[49] Zhao D., Qu Q., Dai H., Liu Y., Jiang L., Huang X., Hao C. (2017). Effects of Hypoxia-inducible Factor-1α on Endometrial Receptivity of Women with Polycystic Ovary Syndrome. Mol. Med. Rep..

[50] Matsumoto L., Hirota Y., Saito-Fujita T., Takeda N., Tanaka T., Hiraoka T., Akaeda S., Fujita H., Shimizu-Hirota R., Igaue S. (2018). HIF2α in the Uterine Stroma Permits Embryo Invasion and Luminal Epithelium Detachment. J. Clin. Investig..

[51] Chen C., Li C., Liu W., Guo F., Kou X., Sun S., Ye T., Li S., Zhao A. (2020). Estrogen-Induced FOS-like 1 Regulates Matrix Metalloproteinase Expression and the Motility of Human Endometrial and Decidual Stromal Cells. J. Biol. Chem..

[52] Bhurke A., Kannan A., Neff A., Ma Q., Laws M.J., Taylor R.N., Bagchi M.K., Bagchi I.C. (2020). A Hypoxia-Induced Rab Pathway Regulates Embryo Implantation by Controlled Trafficking of Secretory Granules. Proc. Natl. Acad. Sci. USA.

[53] Tong J., Lv S., Yang J., Li H., Li W., Zhang C. (2022). Decidualization and Related Pregnancy Complications. Matern.-Fetal Med..

[54] Li B., Jin N., Lu J., Wang M., Wang J., Chen S. (2025). Decreased OGT Attenuates Endometrial Decidualization and Embryo Implantation by Affecting HIF-1α Stability. Mol. Reprod. Devel.

[55] Zhao W., Wang Y., Liu J., Yang Q., Zhang S., Hu X., Shi Z., Zhang Z., Tian J., Chu D. (2023). Progesterone Activates the Histone Lactylation–Hif1α-Glycolysis Feedback Loop to Promote Decidualization. Endocrinology.

[56] Ouyang L., Gao X., Yang R., Zhou P., Cai H., Tian Y., Wang H., Kong S., Lu Z. (2025). SHP2 Regulates the HIF-1 Signaling Pathway in the Decidual Human Endometrial Stromal Cells. Biol. Reprod..

[57] Zuo R.-J., Gu X.-W., Qi Q.-R., Wang T.-S., Zhao X.-Y., Liu J.-L., Yang Z.-M. (2015). Warburg-like Glycolysis and Lactate Shuttle in Mouse Decidua during Early Pregnancy. J. Biol. Chem..

[58] Ma Q., Beal J.R., Bhurke A., Kannan A., Yu J., Taylor R.N., Bagchi I.C., Bagchi M.K. (2022). Extracellular Vesicles Secreted by Human Uterine Stromal Cells Regulate Decidualization, Angiogenesis, and Trophoblast Differentiation. Proc. Natl. Acad. Sci. USA.

[59] Caniggia I., Winter J., Lye S.J., Post M. (2000). Oxygen and Placental Development During the First Trimester: Implications for the Pathophysiology of Pre-Eclampsia. Placenta.

[60] Caniggia I., Mostachfi H., Winter J., Gassmann M., Lye S.J., Kuliszewski M., Post M. (2000). Hypoxia-Inducible Factor-1 Mediates the Biological Effects of Oxygen on Human Trophoblast Differentiation through TGFβ3. J. Clin. Investig..

[61] Rajakumar A., Conrad K.P. (2000). Expression, Ontogeny, and Regulation of Hypoxia-Inducible Transcription Factors in the Human Placenta1. Biol. Reprod..

[62] Genbacev O., Krtolica A., Kaelin W., Fisher S.J. (2001). Human Cytotrophoblast Expression of the von Hippel–Lindau Protein Is Downregulated during Uterine Invasion in Situ and Upregulated by Hypoxia In Vitro. Dev. Biol..

[63] Caniggia I., Winter J.L. (2002). Adriana and Luisa Castellucci Award Lecture 2001 Hypoxia Inducible Factor-1: Oxygen Regulation of Trophoblast Differentiation in Normal and Pre-Eclamptic Pregnancies—A Review. Placenta.

[64] Ietta F., Wu Y., Winter J., Xu J., Wang J., Post M., Caniggia I. (2006). Dynamic HIF1A Regulation During Human Placental Development. Biol. Reprod..

[65] Kenchegowda D., Natale B., Lemus M.A., Natale D.R., Fisher S.A. (2017). Inactivation of Maternal Hif-1α at Mid-Pregnancy Causes Placental Defects and Deficits in Oxygen Delivery to the Fetal Organs under Hypoxic Stress. Dev. Biol..

[66] Chakraborty D., Rumi M.A.K., Konno T., Soares M.J. (2011). Natural Killer Cells Direct Hemochorial Placentation by Regulating Hypoxia-Inducible Factor Dependent Trophoblast Lineage Decisions. Proc. Natl. Acad. Sci. USA.

[67] Anson-Cartwright L., Dawson K., Holmyard D., Fisher S.J., Lazzarini R.A., Cross J.C. (2000). The Glial Cells Missing-1 Protein Is Essential for Branching Morphogenesis in the Chorioallantoic Placenta. Nat. Genet..

[68] Chiang M.-H., Liang F.-Y., Chen C.-P., Chang C.-W., Cheong M.-L., Wang L.-J., Liang C.-Y., Lin F.-Y., Chou C.-C., Chen H. (2009). Mechanism of Hypoxia-Induced GCM1 Degradation. J. Biol. Chem..

[69] Zhu J., Song G., Zhou X., Han T.-L., Yu X., Chen H., Mansell T., Novakovic B., Baker P.N., Cannon R.D. (2022). CD39/CD73 Dysregulation of Adenosine Metabolism Increases Decidual Natural Killer Cell Cytotoxicity: Implications in Unexplained Recurrent Spontaneous Abortion. Front. Immunol..

[70] Yang S.-L., Tan H.-X., Lai Z.-Z., Peng H.-Y., Yang H.-L., Fu Q., Wang H.-Y., Li D.-J., Li M.-Q. (2022). An Active Glutamine/α-Ketoglutarate/HIF-1α Axis Prevents Pregnancy Loss by Triggering Decidual IGF1+GDF15+NK Cell Differentiation. Cell. Mol. Life Sci..

[71] Lu C., Gao R., Qing P., Zeng X., Liao X., Cheng M., Qin L., Liu Y. (2024). Single-Cell Transcriptome Analyses Reveal Disturbed Decidual Homoeostasis in Obstetric Antiphospholipid Syndrome. Ann. Rheum. Dis..

[72] Gao L., Xu Q.-H., Ma L.-N., Luo J., Muyayalo K.P., Wang L.-L., Huang D.-H., Xiao X.-J., Cheng S.-B., Mor G. (2022). Trophoblast-Derived Lactic Acid Orchestrates Decidual Macrophage Differentiation via SRC/LDHA Signaling in Early Pregnancy. Int. J. Biol. Sci..

[73] Qin X.-Y., Shen H.-H., Zhang X.-Y., Zhang X., Xie F., Wang W.-J., Xiong Y., Mei J., Li M.-Q. (2023). Hypoxia-Mediated Chemotaxis and Residence of Macrophage in Decidua by Secreting VEGFA and CCL2 during Normal Pregnancy. Reproduction.

[74] Köstlin-Gille N., Dietz S., Schwarz J., Spring B., Pauluschke-Fröhlich J., Poets C.F., Gille C. (2019). HIF-1α-Deficiency in Myeloid Cells Leads to a Disturbed Accumulation of Myeloid Derived Suppressor Cells (MDSC) During Pregnancy and to an Increased Abortion Rate in Mice. Front. Immunol..

[75] Casazza A., Laoui D., Wenes M., Rizzolio S., Bassani N., Mambretti M., Deschoemaeker S., Van Ginderachter J.A., Tamagnone L., Mazzone M. (2013). Impeding Macrophage Entry into Hypoxic Tumor Areas by Sema3A/Nrp1 Signaling Blockade Inhibits Angiogenesis and Restores Antitumor Immunity. Cancer Cell.

[76] Lin N., Simon M.C. (2016). Hypoxia-Inducible Factors: Key Regulators of Myeloid Cells during Inflammation. J. Clin. Investig..

